# BET Bromodomain Proteins Brd2, Brd3 and Brd4 Selectively Regulate Metabolic Pathways in the Pancreatic β-Cell

**DOI:** 10.1371/journal.pone.0151329

**Published:** 2016-03-23

**Authors:** Jude T. Deeney, Anna C. Belkina, Orian S. Shirihai, Barbara E. Corkey, Gerald V. Denis

**Affiliations:** 1 Department of Medicine, Section of Endocrinology, Obesity Research Center, Evans Biomedical Research Center; Boston University School of Medicine, 650 Albany Street, X804, Boston, Massachusetts 02118, United States of America; 2 Flow Cytometry Core Facility, Boston University School of Medicine, 650 Albany Street, X326, Boston, Massachusetts 02118, United States of America; 3 Department of Pharmacology and Experimental Therapeutics, and Section of Hematology/ Oncology, Cancer Research Center; Boston University School of Medicine, 72 East Concord Street, K520, Boston, Massachusetts 02118, United States of America; University of Alabama at Birmingham, UNITED STATES

## Abstract

Displacement of Bromodomain and Extra-Terminal (BET) proteins from chromatin has promise for cancer and inflammatory disease treatments, but roles of BET proteins in metabolic disease remain unexplored. Small molecule BET inhibitors, such as JQ1, block BET protein binding to acetylated lysines, but lack selectivity within the BET family (Brd2, Brd3, Brd4, Brdt), making it difficult to disentangle contributions of each family member to transcriptional and cellular outcomes. Here, we demonstrate multiple improvements in pancreatic β-cells upon BET inhibition with JQ1 or BET-specific siRNAs. JQ1 (50–400 nM) increases insulin secretion from INS-1 cells in a concentration dependent manner. JQ1 increases insulin content in INS-1 cells, accounting for increased secretion, in both rat and human islets. Higher concentrations of JQ1 decrease intracellular triglyceride stores in INS-1 cells, a result of increased fatty acid oxidation. Specific inhibition of both Brd2 and Brd4 enhances insulin transcription, leading to increased insulin content. Inhibition of Brd2 alone increases fatty acid oxidation. Overlapping yet discrete roles for individual BET proteins in metabolic regulation suggest new isoform-selective BET inhibitors may be useful to treat insulin resistant/diabetic patients. Results imply that cancer and diseases of chronic inflammation or disordered metabolism are related through shared chromatin regulatory mechanisms.

## Introduction

Attention has been focused recently on identification of mutant genomes in human cancer. Chromatin regulators frequently number among the functionally significant alterations that are repeatedly associated with malignancies, and many of these genes encode bromodomain-containing proteins. The bromodomain motif was first described in brahma, a catalytic component of the *Drosophila* SWI/SNF chromatin remodeling complex [1]; it is also present in the yeast transcription factor *BDF1* [2,3] and in *female sterile homeotic (fsh)* [4], a *Drosophila* developmental regulator [5] that functions as an upstream activator of trithorax [6]. These initial papers established the importance of bromodomain proteins [7] in transcriptional co-activation [8,9], histone acetylation [10], cell cycle progression [11,12] and as an effector of signal transduction through the Mediator complex [13,14]. The bromodomain is a 110 amino acid motif comprised of four anti-parallel α-helices with two connecting loops that form a binding pocket for ε-acetyl-lysines of histones present in nucleosomal chromatin [15]. Bromodomains are found in chromatin regulators, transcription factors, co-activators, co-repressors, histone acetylases and related epigenetic factors that control transcription. Double, mutually related bromodomains are found in the four mammalian homologs of *fsh*, called the Bromodomain and ExtraTerminal (BET) family (Brd2, Brd3, Brd4 and Brdt). We reported the first mammalian function of a BET protein [16], showing that Brd2 expression drives *cyclin A* transcription and cell cycle progression [17,18], which promotes lymphoid malignancy [19]. Since then, mammalian BET proteins have been implicated in transcriptional networks that are important for mitosis [20–22], proliferation [23] and acute leukemia [24,25]. Mammalian BET proteins also play central roles in post-mitotic memory [26], virus latency [27,28] and virus episomal persistence [29], adipogenesis [30], learning and memory [31] and inflammation [32,33]. The ubiquity of human BET proteins and their fundamental nature as epigenetic interpreters in numerous cell types suggest that this list is far from comprehensive.

The exciting development of small molecule inhibitors of BET protein binding to chromatin [34,35] has revealed that histone-protein interactions are ‘druggable’ in ways that may benefit cancer patients [36,37]. These inhibitors compete for the acetyl-lysine binding pocket of the bromodomain, and so displace the BET protein from chromatin, which alters the transcriptional activity of the target gene. In the case of certain cancers, in which BET proteins are essential for cell cycle in cooperation with myc, this inhibition blocks proliferation, promotes apoptosis or differentiation [24,25,34,36,37]. Certain of these small molecules have entered clinical trials for malignancies for which the existing therapeutic options have been limited [38–40], such as NUT midline carcinoma [41,42]. Not surprisingly [43], acquired chemoresistance to BET inhibitors may be an emergent problem [44]. Nevertheless, there have been promising signs for combinatorial use of BET inhibitors, most recently in treatment of pancreatic ductal adenocarcinoma [40].

Although recent excitement has centered on the transcriptional co-activator activity of BET proteins, particularly for cell cycle and proliferation genes in cancer, it has long been clear that BET proteins also function as co-repressors of transcription, depending on the signal transduction, cellular and gene context [15,45,46]. Interesting therapeutic opportunities may lie with small molecule inhibition of the co-repressor functions of BET proteins; beneficial, increased adipogenesis has already been reported as an effect of Brd2 knockdown in pre-adipocytes [30]. We implicated Brd2 in obesity and Type 2 diabetes seven years ago, when we published the first paper showing that reduced expression of this BET isoform promotes pancreatic β-cell proliferation and function [30], as well as attenuated inflammation [32] and improved adipogenesis in mouse and tissue culture models [30]. These new developments have prompted a shift in attention beyond chromatin regulator function in malignancy to a broader concern that includes inflammation, obesity and metabolism.

A major concern for the bromodomain inhibitor field is that the first generation small molecule inhibitors are not strongly selective among BET isoforms Brd2/3/4, and, at most reported concentrations, effectively inhibit all family members. Furthermore, each BET protein contains two acetyl-lysine binding bromodomains, each of which is targeted by current inhibitors, with unclear implications for BET protein-associated enzymes. A misunderstanding has arisen regarding the most widely used of these inhibitors, JQ1, which has been thought of as a Brd4 inhibitor. Persistent confusion can be traced to the original JQ1 report [34], which focused attention on Brd4 as an important target. Supplementary data in that report make clear that JQ1 is not highly selective among Brd2 and Brd3; all are very effectively inhibited by the active enantiomer of JQ1. Unfortunately, a cascade of incomplete or misleading reports has followed that refer to JQ1 as a Brd4 inhibitor, without proper controls to show that the non-testis specific isoforms Brd2 and Brd3 are not involved in the functions under study. Because these proteins are not redundant in their functions, pan-BET inhibitors like JQ1 cannot properly be interpreted without specific RNA knockdown or small molecules of proven selectivity. Efforts to develop small molecule inhibitors that are selective for individual BET family proteins continue to advance this area [47].

Indeed, in the present paper, we report that individual BET proteins in the family (Brd2 and Brd4, but not Brd3) control related but distinct functions in the β-cell. We show that inhibition of BET bromodomain proteins actually increases production of insulin and improves pancreatic β-cell function. These results build on our earlier published observations showing that inhibition of BET proteins preserves metabolism in obesity [15,30]. The epigenetic pathways of Brd2 and other BET proteins are not known in the β-cell [48]. A recent report, showing that the small molecule BET inhibitor I-BET151 may have value for Type 1 diabetes [49], has already prompted excitement for potentially expanded clinical implications [50]. We show for the first time that different BET proteins are responsible for different functions in the β-cell. We investigate whether BET protein inhibition confers metabolic protection that may prevent the development of Type 2 diabetes.

## Materials and Methods

### INS-1 cells

INS-1 cells were cultured in RPMI 1640 media supplemented with 1 mM pyruvate, 10 mM HEPES, 50 IU/ml penicillin, 50 μg/ml streptomycin and 10% fetal bovine serum (FBS) and used between passages 60 and 130. We used two strategies to reduce BET protein function in clonal rat pancreatic β-cells (INS-1 832/13). The first was to use the pan BET protein inhibitor JQ1, followed by more selective siRNA knockdown, to assign metabolic functions to specific BET proteins in the pancreatic β-cell.

### Islet isolation and culture

Rat islets were isolated after collagenase digestion as previously described [51]. The protocol was approved by the Boston University Medical Center Institutional Animal Care and Use Committee. Islets were cultured in RPMI 1640 media supplemented with 50 IU/ml penicillin, 50 μg/ml streptomycin and 10% FBS. Groups of 20 islets were incubated with both active JQ1(+) and non-active JQ1(-) enantiomers (400 nM) [34] for 3 days prior to measurement of insulin content. Human islets were obtained from the National Disease Research Interchange (Philadelphia, PA) and were similarly treated. Informed written consent was obtained from next of kin of donors; this activity was reviewed and authorized by the Boston University Medical Center Institutional Review Board, and operates with approval number H-27516.

### Insulin secretion/content

Insulin secretion from INS-1 cells was measured as previously described [51]. INS-1 cells were incubated with both active (+) and non-active (-) JQ1 enantiomers (50–400 nM) for 3 days prior to measurement of insulin secretion and content. Insulin content was measured from cells and islets extracted in 25 mM NaOH plus 0.1% Triton X-100 in phosphate buffered saline (PBS). Insulin was measured using a HTRF insulin assay from CisBio (Bedford, MA).

### RT-qPCR

RNA from INS-1 cells was isolated using an RNeasy kit (Qiagen, Waltham, MA) and quantified as published [30] using 7500 Fast Real-Time PCR System, Power SYBR Green PCR Master Mix (Life Technologies, Grand Island, NY) and Quantitech primers for rat insulin-1 and glyceraldehyde-3-phosphate dehydrogenase (GAPDH) (Qiagen). Melt curve analysis indicated formation of a single product in all cases. Relative mRNA expression levels were determined using ΔCt values and were normalized to GAPDH.

### β-cell lipid stain

INS-1 cells were stained with Nile Red (1 μg/ml) for 10–15 min at 37°C in RPMI media without serum [52]. After stain removal, cells were imaged using a Nikon TE200 fluorescence microscope (20X magnification) equipped with a cooled Olympus DP72 CCD camera and Cellsense imaging software.

### Thin layer chromatography (TLC)

INS-1 cells incubated with 400 nM JQ1 or BET protein specific siRNA were harvested with trypsin and counted prior to lipid extraction. Cell pellets of 1–2 million cells were frozen and stored at -80°C until extraction. Pellets were extracted with chloroform:methanol (CM) 2:1, dried and stored at -80°C prior to lipid separation. Dried lipids were solubilized in CM 2:1 and applied to Silica coated glass TLC plates (Whatman). Lipids were separated using a mobile phase consisting of hexane: diethyl ether: acetic acid (60:38.5:1.5). Triolein and 1,2-dioleoyl-glycerol (Sigma) were used as TG and DG standards. Lipids were detected using the lipid sensitive Nile red dye [53]. Briefly, the dried plate was dipped for 10 seconds in methanol:water (80:20) containing Nile red (8 mg/L). Background fluorescence was quenched with a rapid dip into diluted bleach solution (1:2500 water) and the plate was dried in a 100°C oven for 20 min. Epifluorescence from Nile red stained lipids was detected with 460 nm excitation and >560 nm emission wavelengths, using an Imagequant gel imager (GE Healthcare Life Sciences). Densitometry analysis was performed using Adobe Photoshop CS3 Extended version 10.0.

### Fatty acid oxidation

INS-1 cells were cultured in 24 well plates to a final density of 0.25 x 10^6^ cells per well. Cells were exposed to JQ1 for 72 hours prior to measurement of fatty acid (FA) oxidation. FA oxidation was measured as [^14^C]CO_2_ evolution from [^14^C]oleate. Briefly, cells were incubated with 500 μl/well of modified Krebs buffer containing either 2 mM or 8 mM glucose and 12.5 μM [^14^C]oleate (54 mCi/mmole, Perkin Elmer, Waltham, MA). A 1.5-cm round Whatman filter paper was suspended above each well and the plate was sealed for 2 hrs. After the incubation period, the filter papers were wetted with β-phenylethyl amine followed by acidification of the media with 100 μl/well of 6M H_2_SO_4_. The cell plate remained sealed for an additional hour in order to trap the [^14^C]CO_2_ produced during the incubation period onto the filters. Filter papers were collected and suspended in Ecoscint scintillation fluid (National Diagnostics, Atlanta, GA) and β particle emission was analyzed using a LabLogic 300SL Liquid Scintillation Counter.

### siRNA knockdown of BET proteins

Dharmacon ON-TARGETplus SMARTpool siRNA (20 nM) with DharmaFECT formulation #1 reagent (GE Life Sciences, Pittsburgh, PA) was used for Brd2, Brd3 and Brd4 knockdown [32]. INS-1 cells were seeded in a 24 well culture plate at 25,000 cells/well and, after 3 days, were transfected for 24 hrs. RNA was extracted 72 hrs after start of transfection. Knockdown was estimated from Affymetrix Gene Chip analysis.

### Microarray gene analysis

This study was comprised of 12 Rat Gene 2.0 ST arrays (Affymetrix, Santa Clara, CA), to profile the rat insulinoma line INS-1 832/13, treated either with a non-targeted (NT) control siRNA or with siRNAs directed against Brd2, Brd3 or Brd4 (n = 3 per experimental group). Biotin labeling was performed using the Ambion WT Expression Kit (Life Technologies, Grand Island, NY) according to the manufacturer's protocol, followed by the GeneChip WT Terminal Labeling and Controls Kit (Affymetrix, Santa Clara, CA). The labeled, fragmented DNA was hybridized to the Rat Gene 2.0 ST Array (Affymetrix, Santa Clara, CA) for 18 hours in a GeneChip Hybridization oven 640 at 45°C with rotation (60 rpm). The hybridized samples were washed and stained using an Affymetrix fluidics station 450. After staining, microarrays were immediately scanned using an Affymetrix GeneArray Scanner 3000 7G Plus (Affymetrix, Santa Clara, CA). Raw Affymetrix CEL files were normalized to produce Entrez Gene-identifier-specific expression values using the implementation of the Robust Multiarray Average (RMA) in the affy package in the Bioconductor software suite (version 2.12), using R version 2.15.1 and the Brainarray ragene20strnentrezg R package (version 17.0.0). Array results are presented as log2-transformed, RMA-normalized Entrez Gene expression values.

### Differential expression analysis

One-way analysis of variance (ANOVA). To identify genes, the expression of which changed coordinately with respect to knockdown of at least one target, a one-way analysis of variance (ANOVA) was performed. We identified genes, the expression of which varied more across all groups than within each group. Benjamini-Hochberg False Discovery Rate (FDR) correction [54] was then applied to obtain FDR-corrected p values ('q' values), which represent the probability that a given result is a false positive based on the distribution of all p values on the array. In addition, the FDR q value was also recomputed after removing probesets that were not expressed above the array-wise median value of at least one array. Probesets with low overall expression are more strongly affected by random technical variation and more likely to produce false positive results.

### Clustering analyses

Genes that were significant by one-way ANOVA were arbitrarily broken up into 10 clusters with similar patterns of differential expression, and heatmaps of each cluster were generated. Heatmaps of genes in individual clusters are contained in Supporting Information files (Figs A-J in S1 File, with numerical cluster data in S2 File). In each heatmap, each row corresponds to a gene, and each column corresponds to a sample, and the clusters are color coded by row sidebars. Prior to drawing each heatmap, each gene has been z-score-normalized (to a mean of zero and a standard deviation of one) across all samples in each row. The colors are scaled so that red and blue indicate z-scores of ≥ 2 or ≤ -2, respectively, and white indicates a z-score of 0 (row-wise mean).

### Statistical analysis

Statistical analysis was performed using Student’s t-test where indicated. Values were plotted as averages +/- SEM. *P* <0.05 was considered significant and was denoted by an asterisk (*).

## Results

### JQ1 increases INS-1 insulin secretion and β-cell insulin content

**Fig 1:**
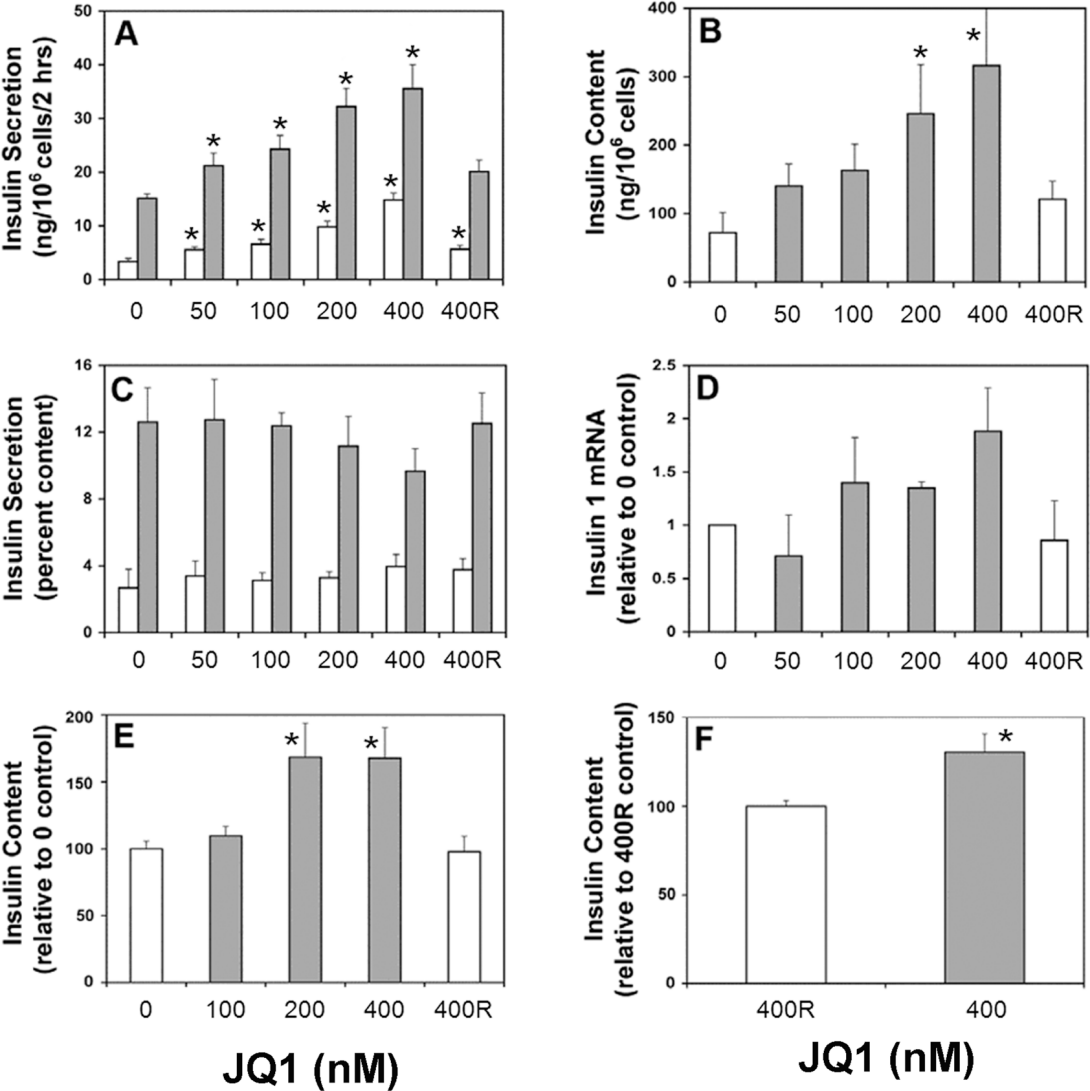
JQ1 increases insulin secretion, content and gene expression in pancreatic β-cells. The rat pancreatic β-cell line INS-1, or isolated islets, were cultured for 3 days as described in Methods, in the presence of indicated concentrations (nM) of JQ1. Except where specified, JQ1 was the active ‘S’ (+) enantiomer [34]. The inactive ‘R’ (-) enantiomer was the negative control. (A) Analysis of insulin secretion in INS-1 cells. Insulin secretion was measured under either basal glucose conditions (2 mM, white bars) or stimulated conditions (8 mM, gray bars). (n = 4) (B) Analysis of insulin content in INS-1 cells. (n = 4) (C) Analysis of insulin secretion, corrected for insulin content, in INS-1 cells. (n = 3) (D) Analysis of *Ins-1* gene expression in INS-1 cells, measured by RT-PCR. (n = 2 separate experiments). (E) Analysis of insulin content in rat islets. (n = 3) (F) Analysis of insulin content in human islets. (3 samples from 2 donors; * p<0.05).

To approach the above questions, we cultured the rat pancreatic β-cell line INS-1 for 3 days with the (+) enantiomer of JQ1 and observed that JQ1 increased insulin secretion (Fig 1A). The effect was dose-dependent, occurred at concentrations as low as 50 nM, and was observed at both 2 mM (basal) glucose and 8 mM (stimulated) glucose. Total insulin content in INS-1 cells was increased over the same JQ1 range (Fig 1B), such that insulin release as a percentage of content was mostly unaffected (Fig 1C). JQ1 increased insulin-1 mRNA over a similar concentration range, as measured with RT-PCR (Fig 1D). To demonstrate BET inhibition in β-cells from intact isolated islets, we cultured small groups of both rat and human islets for 3 days with and without 400 nM JQ1, or with JQ1 enantiomeric control. Insulin content was increased in rat (Fig 1E) and human islets (Fig 1F) exposed to JQ1 compared to controls. This result confirms that effects of JQ1 are not limited to clonal, insulin secreting cells and suggests potential therapeutic benefits to humans.

### JQ1 reduces lipid in INS-1 cells

**Fig 2:**
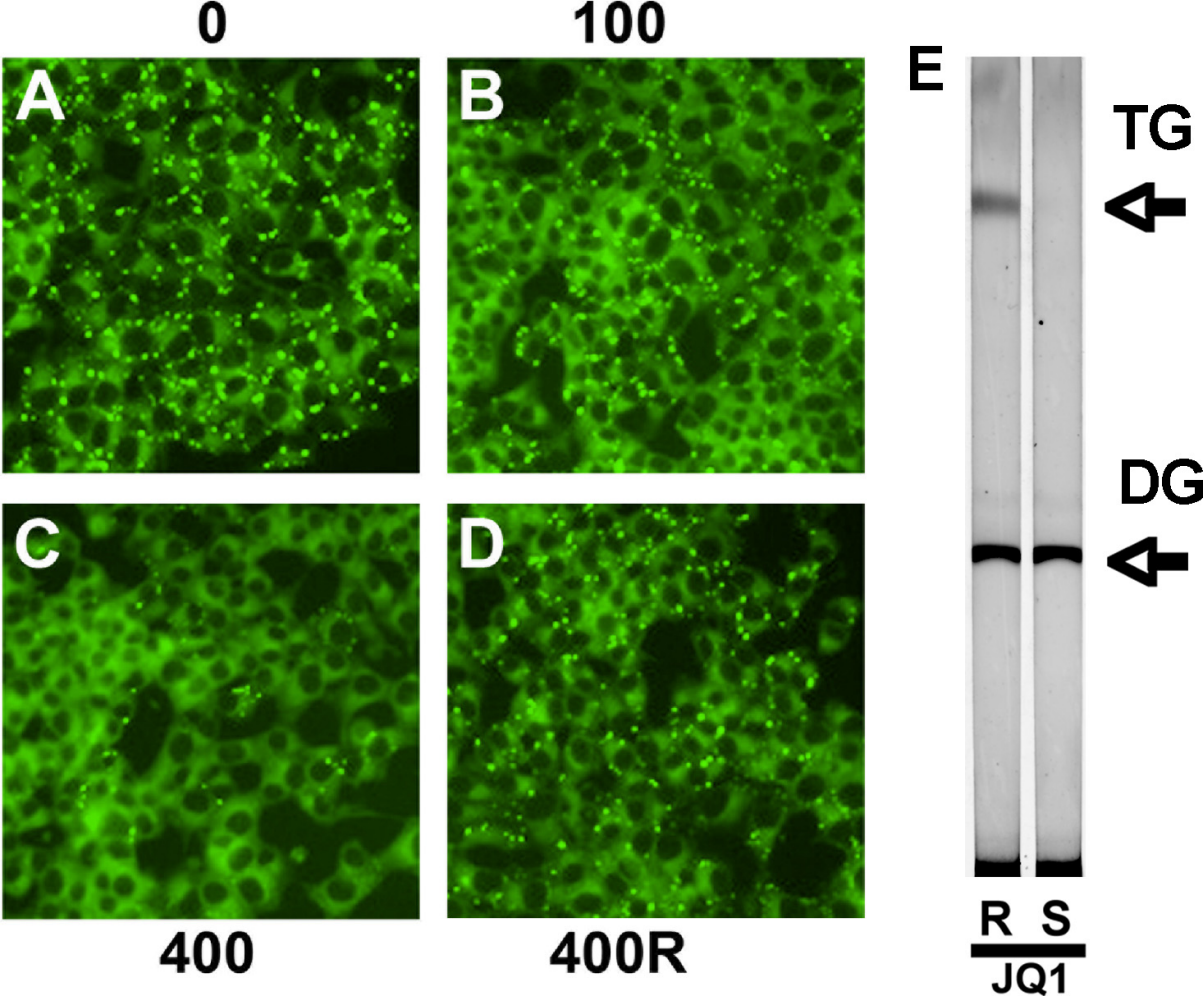
JQ1 reduces intracellular lipid in INS-1 cells. (A) Images are of INS-1 cells incubated with indicated concentrations of JQ1 for 3 days and stained with Nile Red. Results are from a single representative experiment repeated 5 times. Lipid droplets appear as bright dots within cells in micrographs (40X magnification). 0 nM JQ1, control (B) 100 nM JQ1 (active ‘S’ enantiomer) (C) 400 nM JQ1 (active ‘S’ enantiomer) (D) 400 nM JQ1 (inactive ‘R’ enantiomer). (E) Thin layer chromatogram of neutral lipid extracts from INS-1 cells incubated for 3 days with 400 nM of either JQ1 inactive ‘R’ enantiomer or the active JQ1 ‘S’ enantiomer. Triglyceride (TG) and diglyceride (DG) are indicated by arrows.

In parallel, exposure to JQ1 reduced lipid droplets within INS-1 cells in a dose-dependent manner. Low concentration JQ1 (100 nM) had little effect on intracellular lipid stores compared to control (Fig 2A–2B), whereas higher concentrations (400 nM) dramatically depleted lipid droplets (Fig 2C). The inactive enantiomer of JQ1 had no effect at 400 nM (Fig 2D). In order to confirm that Nile red staining of INS-1 cells was providing a measure of intracellular triglyceride (TG) containing lipid droplets, we further examined the ability of JQ1 to reduce TG under our conditions. Results (Fig 2E) show a representative TLC separation of lipid extracts from INS-1 cells incubated with JQ1. Three days’ exposure to 400 nM active JQ1 enantiomer significantly reduced cellular TG by 72 ± 10% (n = 6) compared to the inactive enantiomer, matching Nile red staining of reduced lipid droplets within INS-1 cells. These data confirm the fact that JQ1 alters lipid metabolism in the INS-1 clonal ß-cell line. Loss of intracellular lipids could be the result of a number of affected metabolic pathways including increased lipolysis, decreased triglyceride synthesis or increased FA oxidation.

### JQ1 stimulates FA oxidation

**Fig 3:**
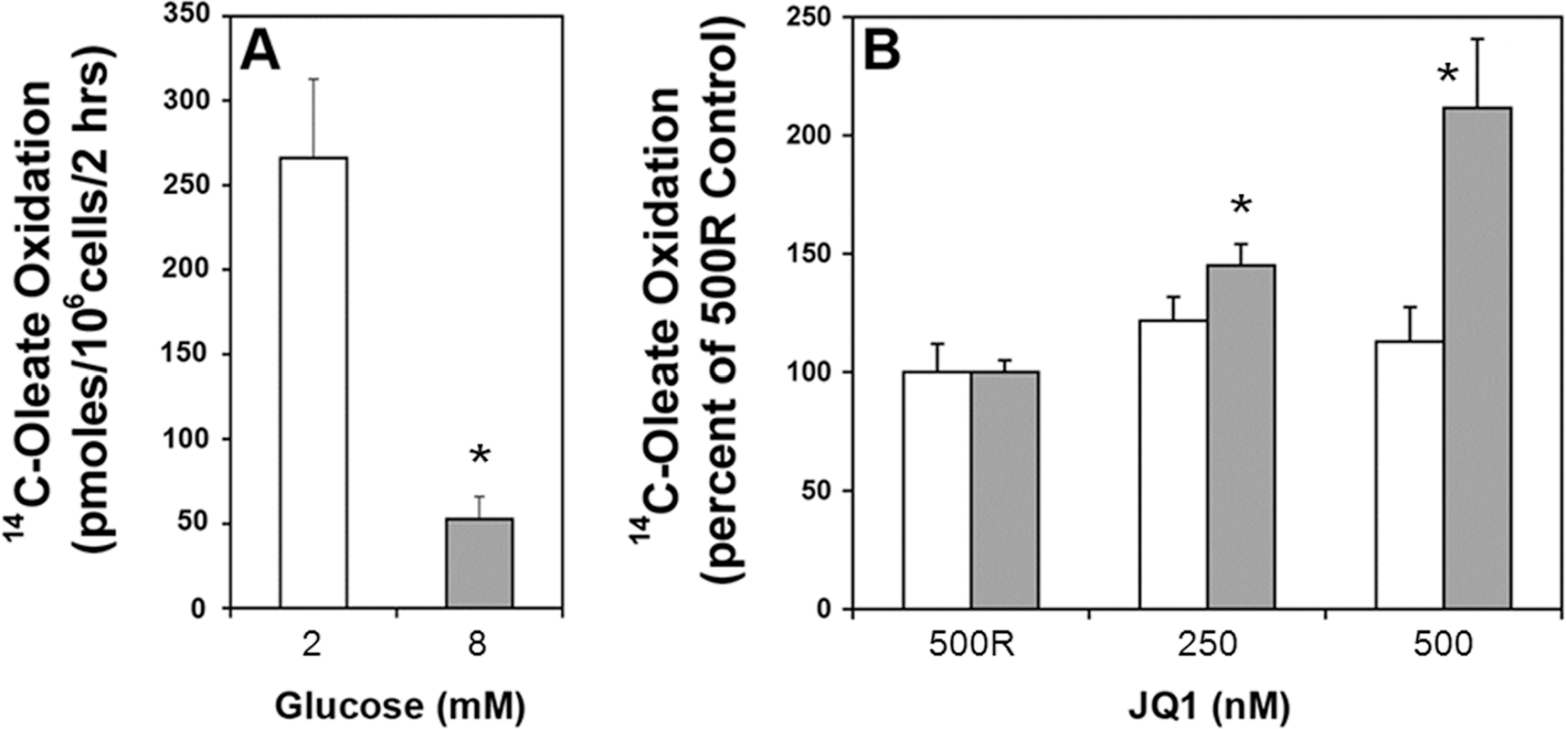
JQ1 reduces the ability of glucose to inhibit fatty acid oxidation in INS-1 cells. (A) Glucose inhibits FA oxidation in INS-1 cells. [^14^C]oleate oxidation, as described in Methods, was measured in INS-1 cells incubated in 2 mM glucose (*white bars*) and 8 mM glucose (*gray bars*). 8 mM glucose inhibits FA oxidation 80% compared to 2 mM glucose control. (B) JQ1 (250–500 nM) increased FA oxidation up to 2-fold in the presence of 8 mM glucose. Oxidation in the presence of inactive JQ1 (R) was set at 100% for both 2 and 8 mM glucose. (A-B n = 3 experiments; * p<0.05).

To determine how BET inhibition decreases lipid accumulation, we first examined the effects of JQ1 on FA oxidation. Increasing glucose from 2 mM to 8 mM inhibited FA oxidation by 80% as expected in INS-1 cells (Fig 3A). Three days’ exposure to JQ1 (250–500 nM) increased FA oxidation at stimulatory glucose, when compared to inactive JQ1(R) control (Fig 3B, *gray bars*), indicating the effect of glucose to inhibit FA oxidation was dampened. Thus, INS-1 cells cultured in RPMI media (11 mM glucose) and JQ1 have a greater capacity to oxidize FA than control, which contributes to loss of intracellular lipid droplets over time (Fig 2). FA oxidation measured at basal 2 mM glucose was unchanged (Fig 3B, *white bars*), suggesting that the FA oxidizing machinery of the cell remained intact.

### siRNA reveals distinct functions of BET proteins in β-cells

**Fig 4:**
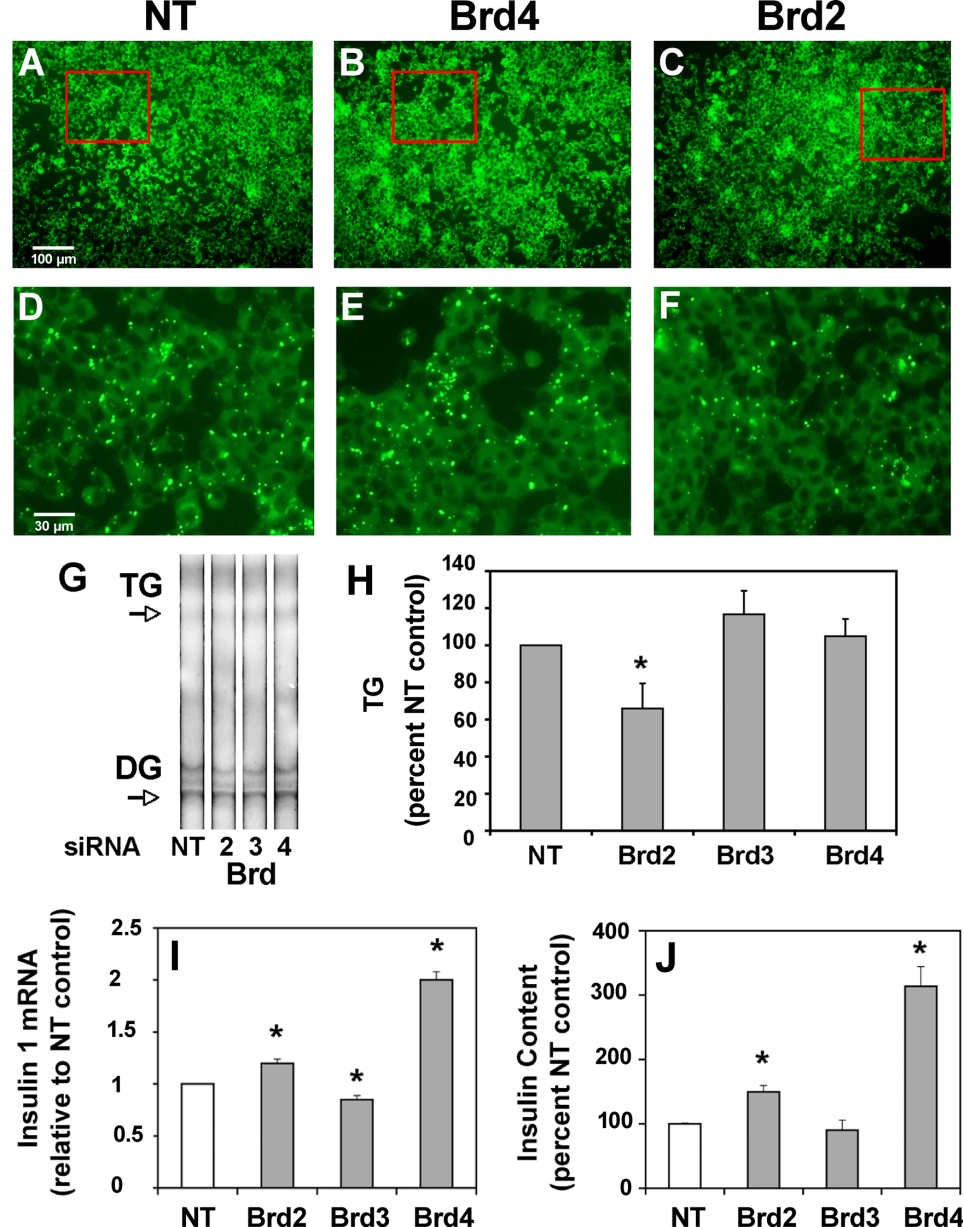
Selective BET inhibition decreases intracellular lipid, alters insulin gene expression and improves insulin production in the pancreatic β-cell. (A) Intracellular lipid droplets were measured in β-cells in the presence of non-targeted (NT) siRNA control, as described in Methods. Results are representative of three separate experiments. (B) Lipid droplets are not reduced in the presence of Brd4-selective siRNA. Results are representative of three separate experiments. (C) Lipid droplets are reduced in the presence of Brd2-selective siRNA. (D, E and F) Enlarged view of cells within red box of panels A, B and C respectively detailing intracellular lipid droplets. Results are representative of three separate experiments. (G) Thin layer chromatogram of neutral lipid extracts from INS-1 cells incubated with BET specific and non-targeted siRNAs. Triglyceride (TG) and diglyceride (DG) are indicated by arrows. Representative of 4 separate experiments. (H) Average densitometry results from TLC plates of 4 separate experiments. Brd2-selective siRNA significantly reduced TG in INS-1 cells compared to Brd3- and Brd4-selective siRNA and NT control (* p<0.05). (I) Brd4 siRNA increased insulin mRNA two-fold compared to the NT control. Brd2 siRNA treatment had less effect than Brd4 siRNA. (Duplicate measures in single experiment) (J) Brd4 siRNA increased insulin content three-fold compared to the NT control. Brd2 siRNA treatment had less effect than Brd4 siRNA. (n = 3 separate experiments; * p<0.05).

The different concentrations of JQ1 required to increase insulin secretion (as low as 50 nM), or deplete lipid stores (200–400 nM), suggested the observed effects are mediated by distinct BET proteins. We used siRNA knockdown to identify the specific BET protein responsible [32]. We did not target Brdt because its expression is testis-restricted. Intracellular lipid droplets were dramatically reduced in INS-1 cells compared to non-targeted siRNA control (Fig 4A and 4D) after Brd2 siRNA treatment (Fig 4C and 4F), whereas incubation with Brd3 siRNA (unpublished observations) or Brd4 siRNA (Fig 4B and 4E) had little effect. We again used TLC to confirm the TG lowering effect of specific siRNAs. Brd2 siRNA significantly reduced TG in INS-1 cells by 34 ± 14% compared to both NT control and Brd4 siRNAs (Fig 4G and 4H). This result identifies for the first time Brd2 as an epigenetic regulator of lipid handling in pancreatic β-cells. These results also rule out the possibility that the lipid reducing effects observed using JQ1 are due to nonspecific interactions with targets other than BET proteins.

Our results for the first time identify Brd4 as a major BET regulator of insulin mRNA (Fig 4F). This mechanism is also reflected in increased insulin content in INS-1 cells (Fig 4J). In comparison, Brd2 siRNA treatment showed a smaller increase in insulin mRNA and content, whereas exposure to Brd3 siRNA did not increase either insulin mRNA or content.

### Microarray analysis of siRNA-induced BET knockdown

Microarray analysis (Rat Gene 2.0 ST, Affymetrix, Santa Clara, CA) revealed siRNA-induced knockdown of all BET targets was achieved; Brd2, Brd3 and Brd4 mRNAs were reduced by 23%, 37% and 26%, respectively, compared to the non-targeted siRNA control. Although not dramatic, knockdowns of individual BET targets were highly selective (Table A in S1 File). Thus, relatively small changes in gene expression of Brd4 and Brd2 resulted in the distinct INS-1 cell phenotypes observed in this study. One-way analysis of variance (ANOVA) identified 1100 genes, the expression of which changed coordinately with respect to knockdown of at least one BET target (Table B in S1 File (expression-filtered FDR q < 0.1)).

**Fig 5:**
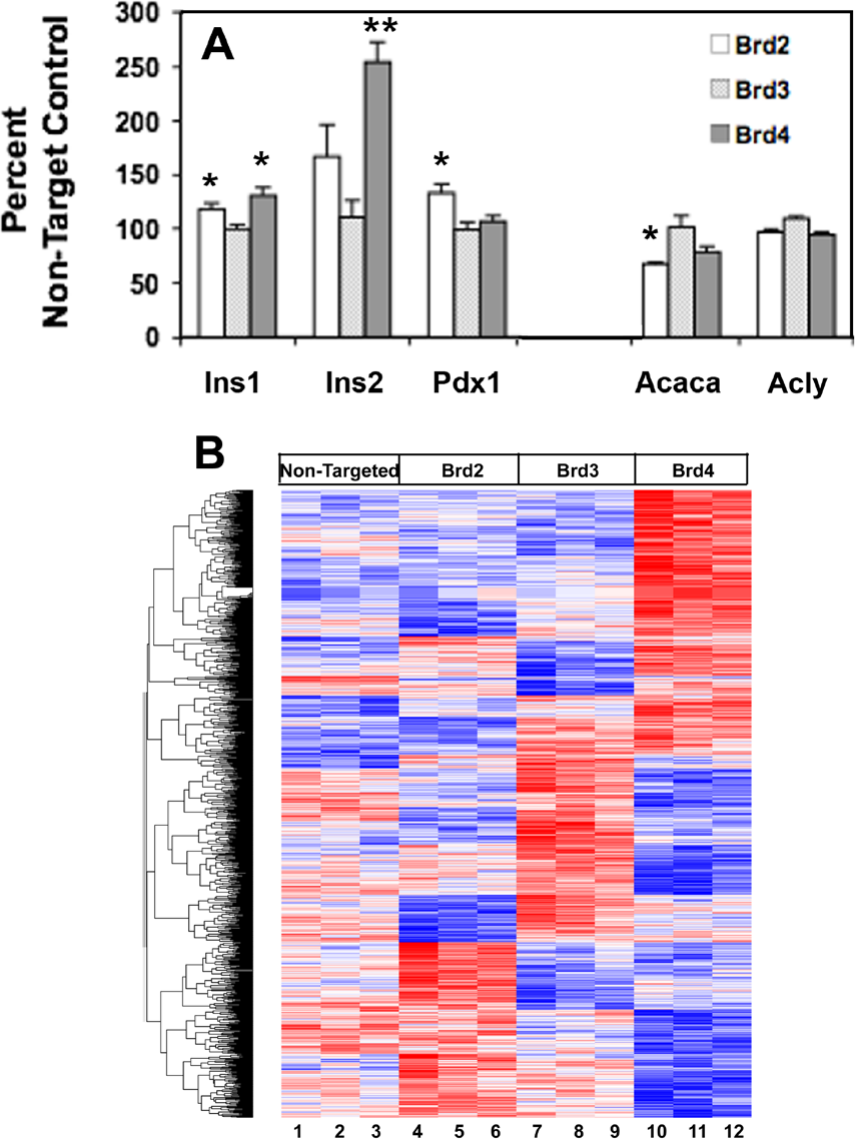
Microarray gene analysis of BET protein specific siRNA treated INS-1 cells. (A) Brd4 siRNA has the greatest effect to increase *Ins1* and *Ins2* gene expression whereas Brd2 siRNA treatment has the greatest effect to reduce *Acaca* gene expression and increase *Pdx1* gene expression, compared to NT control. (n = 3 separate experiments). * p<0.05, ** p<0.001. (B) Heatmap of 1100 genes that were significantly differently expressed compared to the NT control. Red and blue bars represent values that are 2 standard deviations above or below the average of each row (*shown in white*), respectively. Numbered samples were treated as follows: 1–3 with NT control siRNA, 4–6 with Brd2 siRNA, 7–9 with Brd3 siRNA and 10–12 with Brd4 siRNA. Gene identities and hierarchical clustering can be found in S2 File.

Consistent with RT-PCR and total insulin content, microarray gene analysis indicates both *Insulin 1* and *Insulin 2* gene expression levels were increased in β-cells after both Brd2 and Brd4 knockdown, with Brd4 knockdown having the greatest effect. Brd3 knockdown had no effect (Fig 5A, Fig E in S1 File). In addition, Brd2 knockdown increased the gene expression of the insulin transcription factor Pdx1 by 32% (Fig 5A, Fig F in S1 File). This was not the case for Brd4 knockdown, which highlights the potential for multiple roles for BET proteins in regulating insulin gene expression. Brd3 knockdown did not affect gene expression of *Insulin 1*, *Insulin 2* or *Pdx1*.

Malonyl-CoA is known to inhibit carnitine palmitoyl-transferase and FA oxidation in the β-cell and is generated from cytosolic citrate through the sequential actions of ATP citrate lyase (Acly) and acetyl-CoA carboxylase (Acaca). Microarray gene analysis reveals *Acaca* gene expression is significantly reduced (33%) in INS-1 cells with reduced Brd2, supporting the possibility that decreased malonyl-CoA production mediates increased FA oxidation (Fig 5A and S2 File). No change in ATP citrate lyase gene (*Acly)* expression was detected with Brd2 knockdown. These genes were not significantly altered with Brd3 knockdown, although a trend toward reduced *Acaca* was observed with Brd4 knockdown.

Finally, a heat map generated from log_2_ expression values across all samples (Fig 5B) revealed little overlap among 1100 probesets either upregulated or downregulated by individual knockdown of Brd2, Brd3 and Brd4, indicating non-redundant, functional selectivity of BET protein family members in the β-cell. Results (Fig 5B) show clearly that patterns of gene expression cannot be meaningfully interpreted in experiments with currently available pan-BET inhibitors [15], because none of these small molecules are sufficiently isoform-selective.

## Discussion

In this paper, we describe multiple beneficial effects of BET protein inhibition on β-cell function. The first benefit is potentiation of insulin transcription, leading to increased insulin content in INS-1 cells, as well as in both rat and human islets. This is the first report to demonstrate that inhibition of Brd4 leads to potent enhancement of insulin transcription. Interestingly, inhibition of Brd4 had a greater effect to increase insulin content in INS-1 cells than Brd2, which has previously been shown to enhance insulin transcription [48]. How BET proteins affect the chromatin machinery of the insulin locus is still not known. Binding of Brd2/Brd4 may prevent the recruitment of a transcription factor(s) that activates insulin transcription [30].

The second benefit is a shift in β-cell fuel utilization, resulting in increased FA oxidation and decreased intracellular TG stores. Glucose normally inhibits FA oxidation due to anapleurosis in the citric acid cycle. As a result, increased citrate is exported from the mitochondria and converted to cytosolic malonyl-CoA through the sequential actions of ATP citrate lyase and acetyl-CoA carboxylase. Malonyl-CoA then limits FA oxidation by inhibiting carnitine palmitoyl-transferase 1 and FA transport into mitochondria. This mechanism has been proposed to spare long chain acyl-CoA for lipid signaling, potentially acting to amplify insulin secretion from the β-cell [55]. However, when fuels such as glucose and FA are chronically elevated, as is the case in obesity and Type 2 diabetes, β-cells accumulate excess lipid that eventually impairs function, including insulin release. Inhibition of Brd2 dampens the effect of glucose to inhibit FA oxidation, which permits increased FA oxidation, prevents excess lipid accumulation and potentially provides protection from glucolipotoxicity. FA oxidation at low glucose concentration was unaffected, suggesting that Brd2 inhibition specifically alters the metabolic fuel switching mechanism of the β-cell, and not the ability of the cell to oxidize FA normally at low glucose conditions.

The small effect of Brd2 to increase insulin mRNA in INS-1 cells may be in part a consequence of its ability to reduce intracellular lipid dramatically. A reduction in the lipid moiety ceramide, a known inhibitor of insulin transcription, may enhance insulin transcription/content in lipid depleted INS-1 cells [56]. INS-1 cells cultured in 11 mM glucose have elevated lipid stores and only a fraction of the insulin content of normal β-cells [57]. Thus, the low starting level of stored insulin, as well as the positive effects of reducing excess intracellular lipid on insulin mRNA, may both contribute to the exaggerated fold-increase in insulin content in INS-1 cells (Fig 1B) compared to normal β-cells after BET protein inhibition with JQ1 (Fig 1E and 1F). We have recently demonstrated a positive correlation between increased insulin content and reduced intracellular lipid in INS-1 cells [57].

The fact that both insulin transcription and FA oxidation can be regulated using the pan-BET inhibitor JQ1 at different concentrations highlights the complexity of BET regulation with the non-selective small molecules presently available. These are inadequate to map the β-cell pathways regulated by each BET isoform [49]. Targeting single BET proteins for metabolic therapy may be preferred over non-selective inhibition of the entire family of proteins. However, the non-selectivity of JQ1 may be beneficial in its role as an anticancer agent; inhibition of Brd4-driven proliferation [34] could work in combination with a Brd2-driven switch in fuel utilization, favoring FA over glucose oxidation [58].

At high doses of next-generation BET inhibitors, patients with insulin resistance and Type 2 diabetes might benefit from both increased insulin stores and increased β-cell capacity to oxidize FA, protecting the β-cell from excess nutrient-induced damage. Similarly, anti-inflammatory effects of BET inhibitors [32] could lower blood glucose in Type 2 diabetes patients. Thus, the possibility that new, chromatin-directed small molecules could offer benefit for obese, diabetic patients is novel and significant.

Low dose, isoform-selective BET inhibitors might also have therapeutic value for Type 1 diabetes patients to boost largely depleted insulin stores. Our results support the notion that BET inhibitors more selective than JQ1 or I-BET151 [50] may be useful individually or in combination to treat insulin resistant/Type 2 diabetic patients throughout the progression of their disease, once our knowledge of how each BET isoform regulates the relevant pathways is fully developed [15]. We expect some of the epigenetically altered metabolic pathways found in the β-cell to be replicated in other tissues such as muscle, fat and liver, to provide a multi-organ defense to prevent the development or reduce the severity of Type 2 diabetes.

Finally, it is intriguing that differentially increased expression of only three BET proteins could shift epigenetically regulated transcriptional networks in different cell types to reduce insulin production in β-cells [30,48], attenuate adipogenesis in pre-adipocytes [30], increase inflammatory cytokine production in macrophages [32,35] and promote cell cycle progression in diverse cancer cell types [17–19;23–25]. These associations underscore the intriguing hypothesis that inflammatory complications of obesity and Type 2 diabetes may drive certain obesity-associated cancers, working in part through common upregulation of BET proteins [46]. We speculate for instance that BET proteins link unresolved pancreatitis with risk for progression of pancreatic adenocarcinoma. Selective, well tolerated small molecule inhibitors of BET proteins thus may offer an opportunity to address multiple co-morbidities of human obesity, including chronic inflammation, diabetes and cancer.

## Supporting Information

S1 File Supporting Materials and Methods: Supporting Text, Supporting References, Supporting Figs A–J, Supporting Tables A-D. (DOCX)

S2 File Numerical data from Affymetrix microarray experiments, in Excel format. (XLSX)
