# Supplementary material for: BET Bromodomain Proteins Brd2, Brd3 and Brd4 Selectively Regulate Metabolic Pathways in the Pancreatic β-Cell
Source: PLoS One. 2016 Mar 23;11(3):e0151329. doi: 10.1371/journal.pone.0151329 (PMC4805167; doi:10.1371/journal.pone.0151329)
Supplement: S1 File — (DOCX) [file pone.0151329.s001.docx]

**Supporting** **Material**

**BET bromodomain proteins Brd2, Brd3 and Brd4 selectively regulate metabolic pathways in the pancreatic β-cell**

**Jude T. Deeney, Anna C. Belkina, Orian S. Shirihai, Barbara E. Corkey, Gerald V. Denis**

**This file includes:**

Supporting Materials and Methods

Supporting Text

Supporting References

Supporting Figures A–J

Supporting Tables A-D

Materials and Methods

**Microarray gene analysis**

**Normalization and quality assessment**

Affymetrix Rat Gene 2.0 ST CEL files were first normalized to produce gene-level expression values using the Robust Multiarray Average (RMA) [59] within Affymetrix Expression Console (version 1.3.0.187), and array quality was assessed by standard quality metrics computed by Expression Console. The data were then re-normalized to produce gene-level expression values using the implementation of RMA in the *affy* package (version 1.36.1) [60] included within in the Bioconductor software suite (version 2.11) [61] and an Entrez Gene-specific probeset mapping (version 17.0.0) from the Molecular and Behavioral Neuroscience Institute (Brainarray) at the University of Michigan [62,63]. GEO Series GSE6136 was also normalized using RMA and Brainarray Entrez Gene version 17.0.0. Array quality was assessed by computing Relative Log Expression (RLE) and Normalized Unscaled Standard Error (NUSE) using the *affyPLM* Bioconductor package (version 1.34.0) [64]. Principal Component Analysis (PCA) was performed using the *prcomp* R function with expression values that had been normalized across all samples to a mean of zero and a standard deviation of one. Differential gene expression with respect to siRNA treatment group across all samples was assessed by performing a one-way ANOVA computed using the *f.pvalue* function in the *sva* package (version 3.4.0), and the significance of each pairwise comparison between groups (corrected for multiple hypothesis testing) was obtained using Tukey's Honest Significant Difference post-hoc test. Student's two-sample *t* test was performed on the coefficients of linear models created using the *lmFit* function in the *limma* package (version 3.14.4). Correction for multiple hypothesis testing was accomplished using the Benjamini-Hochberg false discovery rate (FDR) [54]. Homologs were identified using HomoloGene (version 67) [65]. All statistical analyses were performed using the R environment for statistical computing (version 2.15.1).

A tab-delimited file (which can be opened in Excel), containing the summarization output from Expression Console, including the log2 (expression) values and all annotation for all probesets (including controls), is available at:

http://microarray.bu.edu/~ctsibio/2014-04-18_Corkey/2014-04-18_Corkey.RMA-GENE-FULL.TXT

**Knockdown efficiency**

To assess the efficiency of the knockdown of each siRNA target, the expression of the Affymetrix probeset corresponding to each target was examined in the corresponding knockdown in comparison with control siRNA. The mean log_2_-scale expression of each target is shown in Table A, with siRNA/target pairs in bold italics, followed by the percentage of expression in the control group, in parentheses (computed in linear space). Brd2, Brd3 and Brd4 mRNA were reduced by 23%, 37% and 26%, respectively, compared to the non-targeted siRNA control (Table A). The knockdown of each specific BET target, although not very robust, was very selective because none of the other BET probesets were knocked down significantly by any given targeted siRNA. Thus, relatively small changes in gene expression of Brd4 and Brd2 resulted in the distinct INS-1 cell phenotypes observed in this study.

**Differential expression analysis**

Table B (left columns) shows the number of genes with a p value below various thresholds, as well as the number of genes expected by chance at each threshold. Similarly, Table B (right columns) also shows the number of genes with a q value below various thresholds, with or without expression filtering.

**Tukey's post-hoc test (Honest Significant Difference)**

To identify genes that vary between each pair of experimental groups, Tukey's post-hoc test was then performed. A test such as this one, which performs multiple hypothesis correction across all inter-group comparisons, is the most appropriate when many such comparisons are performed (in this analysis, there are 6 possible comparisons between all pairs of groups). The numbers of genes with p values below various thresholds for each pairwise comparison are tabulated in Table C. The numbers of genes with FDR q values below various thresholds for each pairwise comparison (with or without expression filtering) are tabulated in Table D.

**Clustering analyses**

Note that the genes are in the same order in the Excel file as in each heatmap. The clusters are shown in Figs A – J.

The cluster membership of each gene is tabulated in the following Supplementary Excel file:

Deeney_et_al_Supplementary_File_2

**Human islets**

Cells were isolated from pancreatic islets obtained from cadavers, at facilities affiliated with the National Disease Research Interchange, with informed written consent of relatives of donors as previously described [66].

**Supplementary References**

59. Irizarry RA, Hobbs B, Collin F, Beazer-Barclay YD, Antonellis KJ, Scherf U, Speed TP (2003) Exploration, normalization, and summaries of high density oligonucleotide array probe level data. Biostatistics 4: 249–264. PubMed: 12925520.

60. [Gautier](http://www.ncbi.nlm.nih.gov/pubmed/?term=Gautier%20L%5BAuthor%5D&cauthor=true&cauthor_uid=14960456) L, [Cope L](http://www.ncbi.nlm.nih.gov/pubmed/?term=Cope%20L%5BAuthor%5D&cauthor=true&cauthor_uid=14960456), [Bolstad](http://www.ncbi.nlm.nih.gov/pubmed/?term=Bolstad%20BM%5BAuthor%5D&cauthor=true&cauthor_uid=14960456) RM, [Irizarry RM](http://www.ncbi.nlm.nih.gov/pubmed/?term=Irizarry%20RA%5BAuthor%5D&cauthor=true&cauthor_uid=14960456) (2004) affy--analysis of Affymetrix GeneChip data at the probe level. [Bioinformatics](http://www.ncbi.nlm.nih.gov/pubmed/?term=14960456) 20: 307–315. PubMed: 14960456.

61. Gentleman RC, Carey VJ, Bates DM, Bolstad B, Dettling M, Dudoit S, et al. (2004) Bioconductor: open software development for computational biology and bioinformatics. Genome Biology 5: R80. PubMed: 15461798.

62. Dai M, Wang P, Boyd AD, Kostov G, Athey B, Jones EG, et al. (2005) Evolving gene/transcript definitions significantly alter the interpretation of GeneChip data. Nucleic Acids Res 33: e175. PubMed:16284200.

63. <http://brainarray.mbni.med.umich.edu/Brainarray/Database/CustomCDF>

64. Brettschneider J, Collin F, Bolstad BM, Speed TP. (2008) Quality assessment for short oligonucleotide microarray data. Technometrics 50: 241.

65. NCBI Resource Coordinators (2013) Database resources of the National Center for Biotechnology Information. Nucleic Acids Res 41: D8–D20.

66. Wikstrom JD, Sereda SB, Stiles L, Elorza A, Allister EM, Neilson A, Ferrick DA, Wheeler MB, Shirihai OS (2012) A novel high-throughput assay for islet respiration reveals uncoupling of odent and human islets. PLoS One 7: e33023. doi: 10.1371/journal.pone.0033023.

PubMed: 22606219.


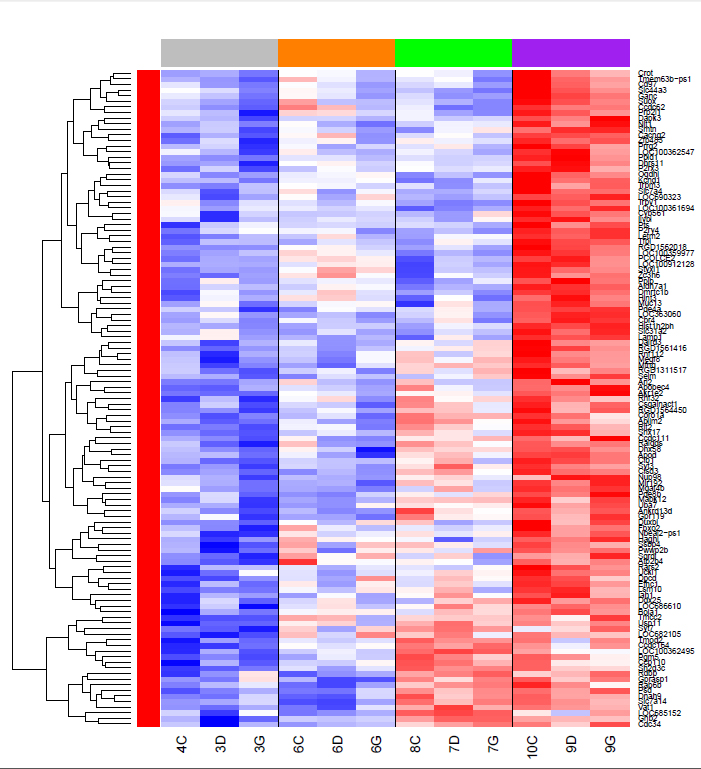
**Figure A. Hierarchical clustering. Cluster 1.**

1 2 3 4 5 6 7 8 9 10 11 12

NT

siBrd3

siBrd2

siBrd4

Cluster 1 contains 117 features; siRNA FDR q expression filter < 0.1

*Gray*, non−targeted; *orange*, Brd2; *green*, Brd3; *purple*, Brd4. Colors are scaled by row/gene (blue/white/red, below/at/above average, respectively). For numerical data, refer to: Deeney_et_al_Supplementary_File_2

**
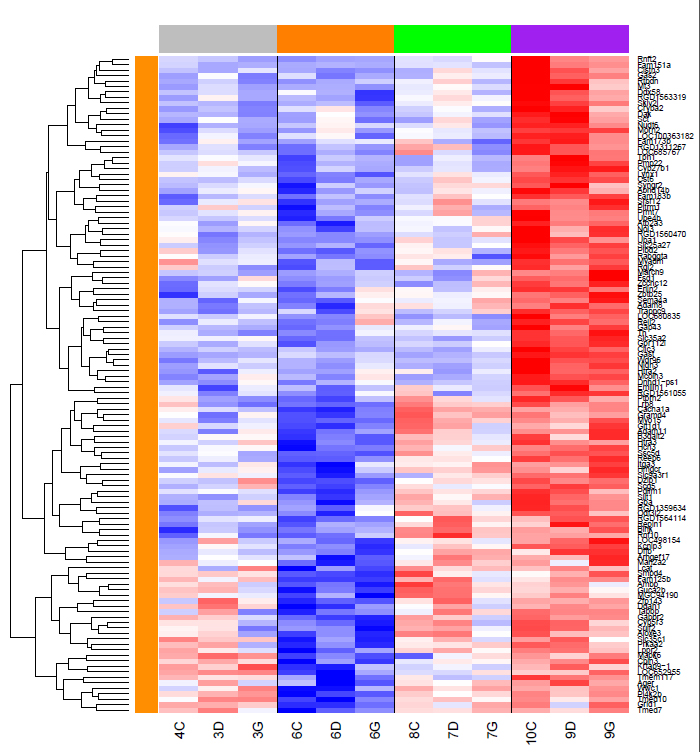
**

siBrd4

siBrd3

siBrd2

NT

**Figure B. Hierarchical clustering. Cluster 2.**

1 2 3 4 5 6 7 8 9 10 11 12

Cluster 2 contains 120 features; siRNA FDR q expression filter < 0.1

*Gray*, non−targeted; *orange*, Brd2; *green*, Brd3; *purple*, Brd4. Colors are scaled by row/gene (blue/white/red, below/at/above average, respectively). For numerical data, refer to: Deeney_et_al_Supplementary_File_2

**
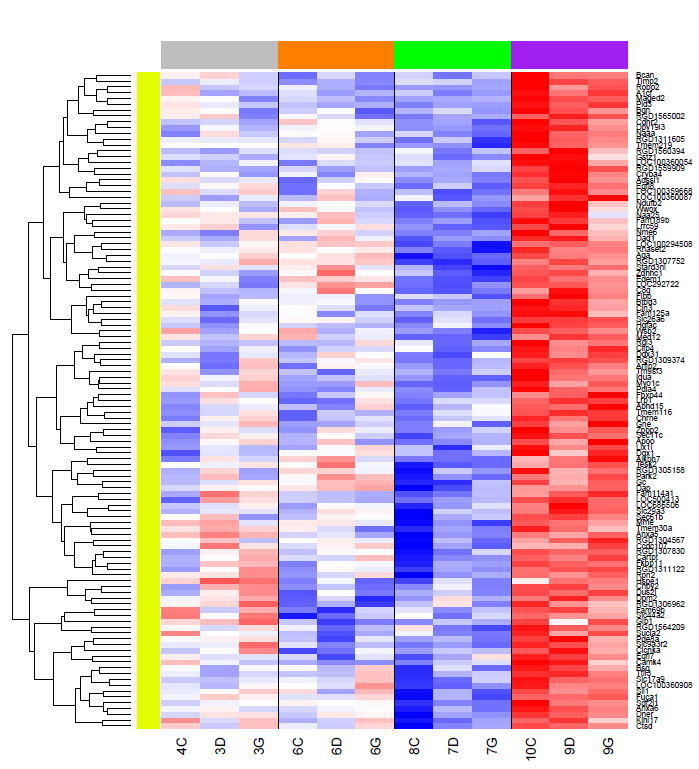
**

siBrd4

siBrd3

siBrd2

NT

**Figure C. Hierarchical clustering. Cluster 3.**

1 2 3 4 5 6 7 8 9 10 11 12

Cluster 3 contains 113 features; siRNA FDR q expression filter < 0.1

*Gray*, non−targeted; *orange*, Brd2; *green*, Brd3; *purple*, Brd4. Colors are scaled by row/gene (blue/white/red, below/at/above average, respectively). For numerical data, refer to: Deeney_et_al_Supplementary_File_2

**
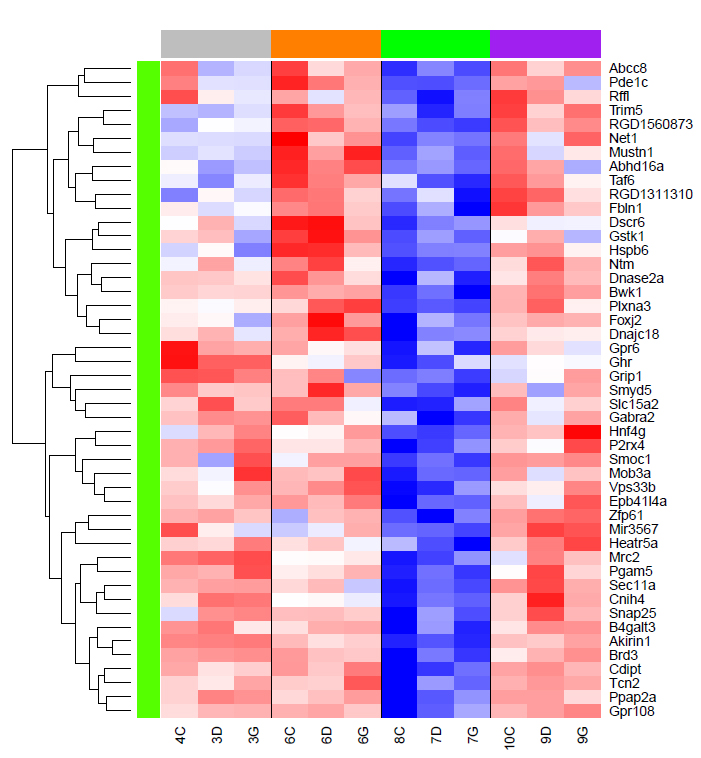
**

NT

siBrd3

siBrd2

siBrd4

**Figure D. Hierarchical clustering. Cluster 4.**

1 2 3 4 5 6 7 8 9 10 11 12

Cluster 4 contains 47 features; siRNA FDR q expression filter < 0.1

*Gray*, non−targeted; *orange*, Brd2; *green*, Brd3; *purple*, Brd4. Colors are scaled by row/gene (blue/white/red, below/at/above average, respectively). Note selectivity of Brd3 knockdown (*arrow*). For numerical data, refer to: Deeney_et_al_Supplementary_File_2

**
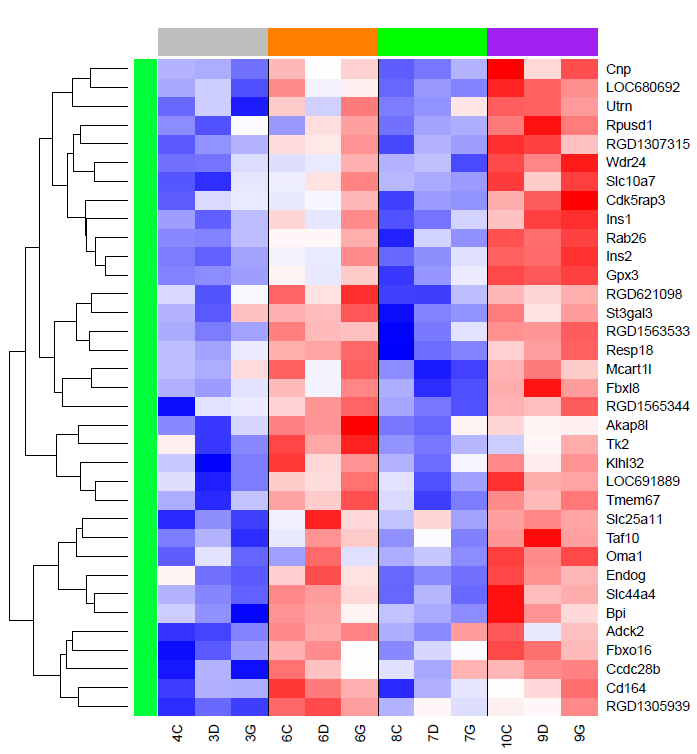
**

siBrd3

siBrd2

NT

siBrd4

**Figure E. Hierarchical clustering. Cluster 5.**

1 2 3 4 5 6 7 8 9 10 11 12

Cluster 5 contains 35 features; siRNA FDR q expression filter < 0.1

*Gray*, non−targeted; *orange*, Brd2; *green*, Brd3; *purple*, Brd4. Colors are scaled by row/gene (blue/white/red, below/at/above average, respectively). Note upregulation of insulin genes *Ins1* and *Ins2* in Brd4 knockdown INS-1 cells compared to Brd2 and Brd3 knockdown (*arrow*). For numerical data, refer to: Deeney_et_al_Supplementary_File_2

**
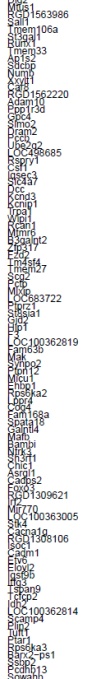

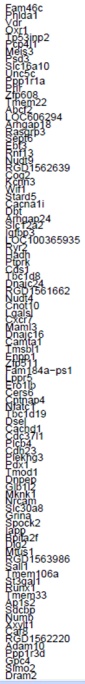

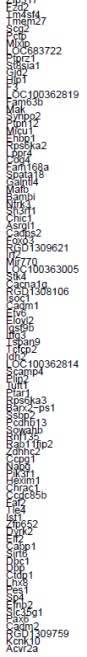

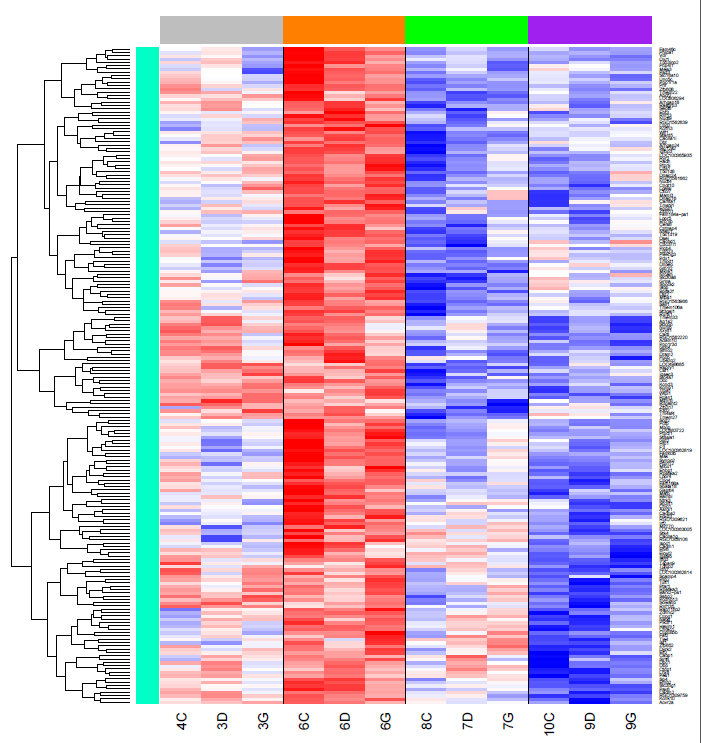
**

siBrd3

siBrd2

NT

siBrd4

**Figure F. Hierarchical clustering. Cluster 6.**

1 2 3 4 5 6 7 8 9 10 11 12

Cluster 6 contains 198 features; siRNA FDR q expression filter < 0.1

*Gray*, non−targeted; *orange*, Brd2; *green*, Brd3; *purple*, Brd4. Colors are scaled by row/gene (blue/white/red, below/at/above average, respectively). Note upregulation of *Pdx1* in Brd2 knockdown INS-1 cells compared to Brd3 and Brd4 (*arrow*). For numerical data, refer to: Deeney_et_al_Supplementary_File_2

**
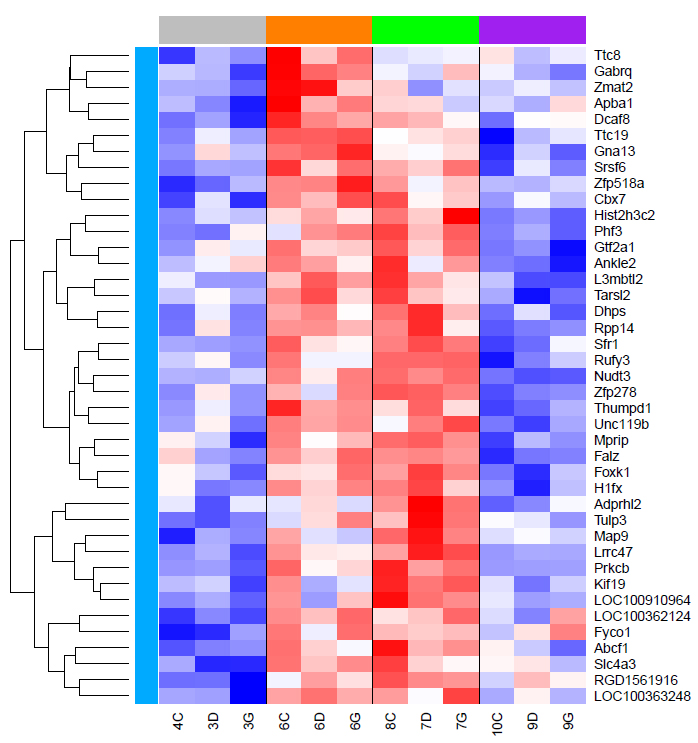
Figure G. Hierarchical clustering. Cluster 7.**

1 2 3 4 5 6 7 8 9 10 11 12

NT

siBrd3

siBrd2

siBrd4

Cluster 7 contains 41 features; siRNA FDR q expression filter < 0.1

*Gray*, non−targeted; *orange*, Brd2; *green*, Brd3; *purple*, Brd4. Colors are scaled by row/gene (blue/white/red, below/at/above average, respectively). For numerical data, refer to: Deeney_et_al_Supplementary_File_2

**
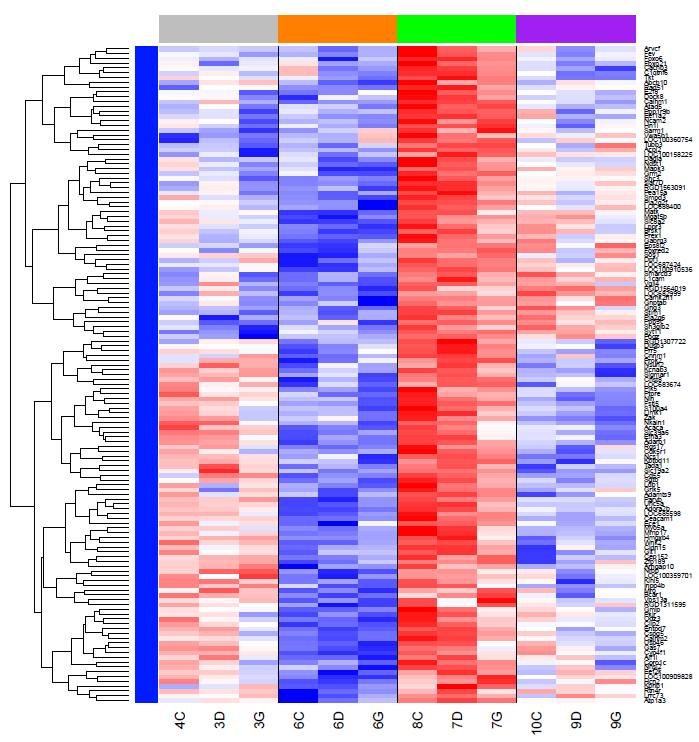
Figure H. Hierarchical clustering. Cluster 8.**

siBrd3

siBrd2

NT

siBrd4

Cluster 8 contains 137 features; siRNA FDR q expression filter < 0.1

1 2 3 4 5 6 7 8 9 10 11 12

*Gray*, non−targeted; *orange*, Brd2; *green*, Brd3; *purple*, Brd4. Colors are scaled by row/gene (blue/white/red, below/at/above average, respectively). For downregulation of *Acaca* in Brd2/4 knockdown INS-1 cells, but upregulation in Brd3 knockdown, please refer to numerical data: Deeney_et_al_Supplementary_File_2

**
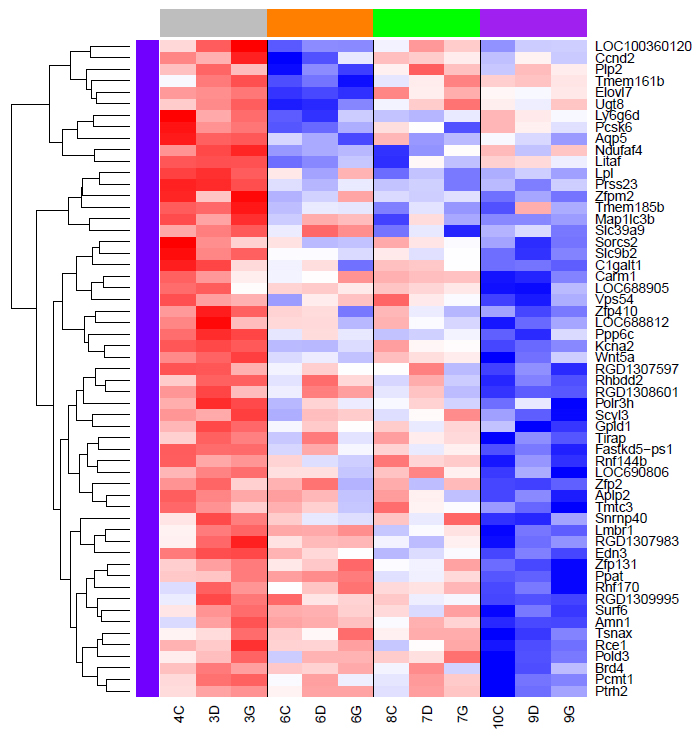
**

siBrd3

siBrd2

NT

siBrd4

1 2 3 4 5 6 7 8 9 10 11 12

**Figure I. Hierarchical clustering. Cluster 9.**

Cluster 9 contains 57 features; siRNA FDR q expression filter < 0.1

*Gray*, non−targeted; *orange*, Brd2; *green*, Brd3; *purple*, Brd4. Colors are scaled by row/gene (blue/white/red, below/at/above average, respectively). Note selectivity of Brd4 knockdown (*arrow*). For numerical data, refer to: Deeney_et_al_Supplementary_File_2

**
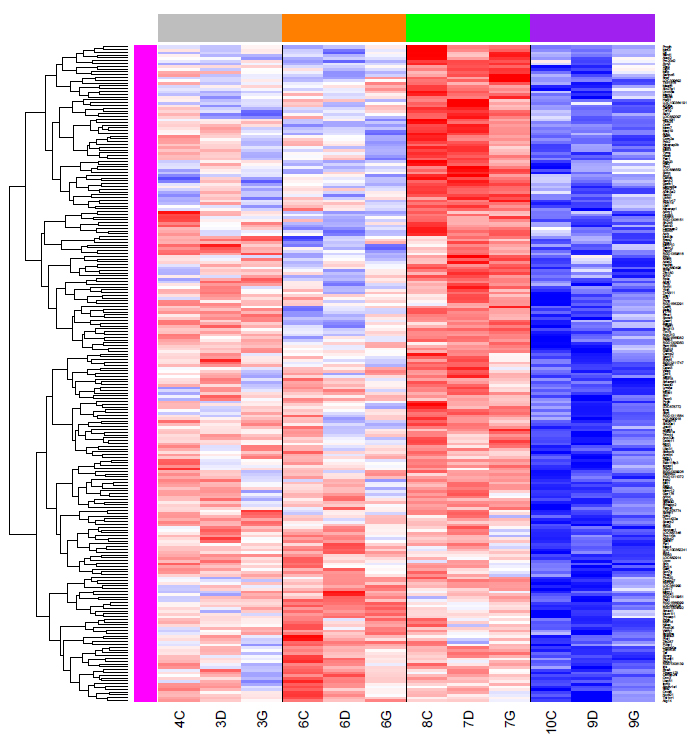
**

siBrd3

siBrd2

NT

siBrd4

**Figure J. Hierarchical clustering. Cluster 10.**

1 2 3 4 5 6 7 8 9 10 11 12

Cluster 10 contains 235 features; siRNA FDR q expression filter < 0.1

*Gray*, non−targeted; *orange*, Brd2; *green*, Brd3; *purple*, Brd4. Colors are scaled by row/gene (blue/white/red, below/at/above average, respectively). For numerical data, refer to: Deeney_et_al_Supplementary_File_2

Table A. Selective knockdown of Brd2, Brd3 and Brd4.

| **siRNA** | | | | |
| --- | --- | --- | --- | --- |
| **Target** | **NT control** | **Brd2** | **Brd3** | **Brd4** |
| Brd2 | 9.6 | ***9.2 (77%)*** | 9.6 (100%) | 9.6 (98%) |
| Brd3 | 8.5 | 8.4 (93%) | ***7.9 (63%)*** | 8.4 (94%) |
| Brd4 | 9.2 | 9.2 (96%) | 9.1 (92%) | ***8.8 (74%)*** |

The knockdown of all targets was relatively weak (reduction to between 63% and 77% of control levels) but selective (none of the other BET probesets were knocked down by a given siRNA).

Table B. Genes with differential expression by one-way ANOVA across all 4 groups.

| **p threshold** | **expected** | **siRNA** | **q threshold** | **all genes** | **expression filter** |
| --- | --- | --- | --- | --- | --- |
| 0.05 | 1017 | 3365 | 0.25 | 2617 | 3077 |
| 0.01 | 203 | 1312 | 0.1 | 658 | 1100 |
| 0.005 | 102 | 867 | 0.05 | 251 | 453 |
| 0.001 | 20 | 331 | 0.01 | 6 | 21 |

There is strong differential expression between at least two siRNA treatment conditions, and removal of probesets with low overall expression substantially improved the FDR q values.

Table C. Nominal p values for pairwise comparisons between experimental groups.

| **p threshold** | **expected** | **Brd2 vs non-targeted** | **Brd3 vs non-targeted** | **Brd4 vs non-targeted** | **Brd3 vs Brd2** | **Brd4 vs Brd2** | **Brd4 vs Brd3** |
| --- | --- | --- | --- | --- | --- | --- | --- |
| 0.05 | 1017 | 598 | 610 | 1140 | 1025 | 1488 | 1483 |
| 0.01 | 203 | 154 | 168 | 427 | 381 | 579 | 555 |
| 0.005 | 102 | 77 | 102 | 289 | 261 | 360 | 366 |
| 0.001 | 20 | 20 | 33 | 103 | 89 | 139 | 144 |

FDR correction was then used to correct for multiple hypotheses across all genes (note that the Tukey and FDR multiple hypothesis corrections are different as they operate across groups and across genes, respectively).

Table D. FDR q values for all pairwise comparisons, both before and after applying the expression filter.

| all genes -------------------------------------- | | | | | | | expression filter------------------------------------ | | | | | | |
| --- | --- | --- | --- | --- | --- | --- | --- | --- | --- | --- | --- | --- | --- |
| **q threshold** | **Brd2 vs NT** | **Brd3 vs NT** | **Brd4 vs NT** | **Brd3 vs Brd2** | **Brd4 vs Brd2** | **Brd4 vs Brd3** | | **Brd2 vs NT** | **Brd3 vs NT** | **Brd4 vs NT** | **Brd3 vs Brd2** | **Brd4 vs Brd2** | **Brd4 vs Brd3** |
| 0.25 | 3 | 1 | 193 | 91 | 308 | 325 | | 8 | 11 | 314 | 243 | 558 | 578 |
| 0.1 | 0 | 1 | 37 | 27 | 86 | 68 | | 1 | 1 | 78 | 48 | 142 | 177 |
| 0.05 | 0 | 1 | 7 | 7 | 21 | 28 | | 0 | 1 | 23 | 20 | 50 | 58 |
| 0.01 | 0 | 1 | 2 | 0 | 3 | 2 | | 0 | 1 | 2 | 0 | 3 | 3 |

As expected from the PCA plot, there was relatively weak differential gene expression between groups compared to non-targeted (NT), except for comparisons with the Brd4 knockdown. Furthermore, removal of probesets with low expression substantially improved the FDR q values.
